# C-Type Lectin-Like Molecule-1 as a Biomarker for Diagnosis and Prognosis in Acute Myeloid Leukemia: A Preliminary Study

**DOI:** 10.1155/2021/6643948

**Published:** 2021-03-11

**Authors:** Jinghua Wang, Weida Wang, Hao Chen, Wenmin Li, Tian Huang, Weiya Zhang, Wei Ling, Peilong Lai, Yulian Wang, Suxia Geng, Minming Li, Xin Du, Jianyu Weng

**Affiliations:** ^1^Department of Hematology, Guangdong Provincial People's Hospital, Guangdong Academy of Medical Sciences, Guangzhou, China; ^2^Department of Hematology Oncology, State Key Laboratory of Oncology in South China, Collaborative Innovation Center for Cancer Medicine, Sun Yat-sen University Cancer Center, Guangzhou, China; ^3^Department of Gastroenterology, Guangdong Provincial People's Hospital, Guangdong Academy of Medical Sciences, Guangzhou, China; ^4^Department of Laboratory Medicine, Guangdong Provincial People's Hospital, Guangdong Academy of Medical Sciences, Guangzhou, China

## Abstract

**Objective:**

AML is a heterogeneous disease both in genomic and proteomic backgrounds, and variable outcomes may appear in the same cytogenetic risk group. Therefore, it is still necessary to identify new antigens that contribute to diagnostic information and to refine the current risk stratification.

**Methods:**

The expression of C-type lectin-like molecule-1 (CLL-1) in AML blasts was examined in 52 patients with newly diagnosed or relapsed/refractory AML and was compared with two other classic markers CD33 and CD34 in AML, in order to assess the value of CLL-1 as an independent biomarker or in combination with other markers for diagnosis in AML. Subsequently, the value of CLL-1 as a biomarker for prognosis was assessed in this malignant tumor.

**Results:**

The results showed that CLL-1 was expressed on the cell surface of the majority of AML blasts (78.8%) and also expressed on leukemic stem cells in varying degree but absent on normal hematopoietic stem cells. Notably, CLL-1 was able to complement the classic markers CD33 or CD34. After dividing the cases into CLL-1^high^ and CLL-1^low^ groups according to cutoff 59.0%, we discovered that event-free survival and overall survival (OS) of the CLL-1^low^ group were significantly lower than that of the CLL-1^high^ group, and low CLL-1 expression seems to be independently associated with shorter OS.

**Conclusions:**

These preliminary observations identified CLL-1 as a biomarker for diagnosis and prognosis of AML.

## 1. Introduction

Acute myeloid leukemia (AML) is the most common form of acute leukemia in adults, which is characterized by the accumulation of immature myeloid cells in the bone marrow (BM) leading to hematopoietic dysfunction [1]. AML is a heterogeneous disease both in clinical manifestation and reaction to therapy. Underlying this is a similar heterogeneity of genomic and proteomic backgrounds, with respect to the latter which is reflected in the immunophenotype of leukemia blasts [2]. Evidence in the literature suggests that the leukemia-associated immunophenotype varies greatly from patient to patient and is not necessarily stable throughout the course of the disease [3]. Therefore, it is still necessary to identify new antigens that contribute to diagnostic information. Cytogenetic and molecular genetic abnormalities are considered to be important factors affecting the prognosis of AML and are increasingly guiding the risk stratification and treatment selection of AML [4]. However, patients in the same risk group may show variable outcomes with a considerable degree of heterogeneity. Consequently, additional prognostic markers are needed to refine the current risk stratification.

Human C-type lectin-like molecule-1 (CLL-1; also known as CLEC12A, CD371, myeloid inhibitory C-type lectin-like receptor (MICL), dendritic cell-associated lectin 2 (DCAL2), or killer cell lectin like receptor-1 (KLRL1)) is emerging as a surface marker of blasts in AML these years. It is a type II transmembrane glycoprotein which plays a role in immune regulation as an inhibitory receptor [5–10], though the ligand of this receptor needs to be determined. The physiological expression of CLL-1 is restricted in hematopoietic cells; it is present on almost all the granulocytes and monocytes and on parts of myeloid progenitors in normal BM and peripheral blood (PB), while absent in nonhematological tissues. Quite apart from that, CLL-1 is lack of expression on normal hematopoietic stem cells (HSCs) in the CD34^+^/CD38^–^ compartment. In the setting of AML, CLL-1 is detected on the majority of AML blasts varying from 77.5% to 92% at diagnosis. More importantly, CLL-1 is also present on leukemic stem cells (LSCs), which is the main cause of treatment failure and leukemia relapse [5, 11–15]. Accordingly, its differential characteristics may make CLL-1 a useful tool for diagnosis and follow-up settings and also an ideal therapeutic target for AML. Indeed, therapeutic use of monoclonal antibody or chimeric antigen receptor-modified T cell targeting CLL-1 has demonstrated to be effective in reducing AML burden in preclinical and clinical trials [14, 16–23]. However, the relationship between the expression of CLL-1 and other AML classical markers is still unclear, and the prognostic value of CLL-1 expression in AML patients is still rarely reported.

In this study, we first examined the expression of CLL-1 in samples of newly diagnosed or relapsed/refractory AML patients and compared it with other classic markers in AML, in order to assess the value of CLL-1 as an independent biomarker or in combination with other markers for diagnosis in AML and subsequently assessed the value of CLL-1 as a biomarker for prognosis in this malignant tumor.

## 2. Materials and Methods

### 2.1. Patient Enrollment and Characteristics

From April 2015 to March 2020, a total of 52 prospectively accrued diagnostic patients with de novo or relapsed AML from the Department of Hematology, Guangdong General Hospital, and Sun Yat-sen University Cancer Center were enrolled in this study. The patients who simultaneously suffered from other malignant tumor were excluded from this study. All samples and medical information were collected with informed patient consent and in accordance with the Declaration of Helsinki. Diagnosis of patients was based on morphology using the French-American-British (FAB) classification, immunophenotyping, cytogenetics, and molecular genetics. Relapse risk for acute promyelocytic leukemia (APL) was classified according to Sanz score [24]. Genetic risk for non-APL AML was classified according to 2017 European LeukemiaNet recommendations [25]. The complete response (CR) was defined as BM blasts < 5%; absence of circulating blasts and blasts with Auer rods; absence of extramedullary leukemia; absolute neutrophil count > 1.0 × 10^9^/L; platelet count > 100 × 10^9^/L. This study was approved by the Ethics Committee of the Guangdong General Hospital and Sun Yat-sen University Cancer Center.

### 2.2. Sample Preparation

BM samples were collected at diagnosis after informed consent from 52 AML patients. In two cases, BM was not available, and PB was used. Five control normal BM samples were obtained from healthy volunteers in the setting of BM harvest for allogeneic BM transplantation after informed consent. The majority of the samples were analyzed freshly. Red blood cells were lysed afterward by 10 minutes of incubation on ice, using 10 mL lysis buffer (155 mM NH_4_Cl, 10 mM KHCO_3_, 0.1 mM Na_2_EDTA, pH 7.4), and washed with phosphate-buffered saline (PBS) containing 0.1% fetal calf serum (FBS). The frozen samples were prepared using red blood cell lysis buffer, then frozen in RPMI 1640 media (Gibco, Grand Island, NY, USA) containing 20% FBS and 10% dimethylsulfoxide and subsequently stored in liquid nitrogen. When analysis was needed, the cells were thawed and suspended in prewarmed RPMI with 10% FBS at 37°C and enabled to recover for 45 min. The prepared samples were then used to perform multiparameter flow cytometry (FCM) subsequently.

### 2.3. Flow Cytometry

Cells were stained with appropriate fluorochrome-conjugated anti-human monoclonal antibodies as follows: CD45-PerCP, CD34-FITC, CD38-PE/cy7, CD33-PE, and CLL1-APC. All the antibodies were purchased from Biolegend (San Diego, CA, USA). PBS was used as a negative control, because for these specific antibodies, isotype controls provided the same results. The gating strategy has been described in detail before [14]. In short, the blast cell population was gated based on low side scatter versus CD45^dim^ expression. For the LSCs subsets, CD34^+^ blasts were identified in blasts, and then, CD34^+^CD38^−^ compartment was defined (Supplementary Figure 1(A, B)). FCM were performed on a BD Fortesa FCM, and results were analyzed using the software FlowJo7.6.5.

### 2.4. Statistical Analysis

We evaluated the predictive value of CLL-1 expression level using the area under the receiver-operating characteristic (ROC) curve and determined the cutoff point to maximize the sum of the sensitivity and specificity. Categorical variables were compared using the chi-square test. The Student's *t* test (two tailed) was used for continuous variables. Survival analysis was performed using the Kaplan-Meier method, and survival between groups was compared using log-rank tests. Univariate and multivariate analyses were used to estimate the prognostic impact of different variables in overall survival (OS) and event-free survival (EFS) using the Cox regression model. OS was measured from the date of diagnosis to the date of death from any cause or the last follow-up. EFS was measured from the date of diagnosis to the date of disease progression or relapse or death from any cause or the last follow-up. Analyses were performed using SPSS version 19 statistical software, and two-tailed values of *p* < 0.05 were considered significant.

## 3. Result

### 3.1. Validation of CLL-1 as an AML Marker

**Figure 1:**
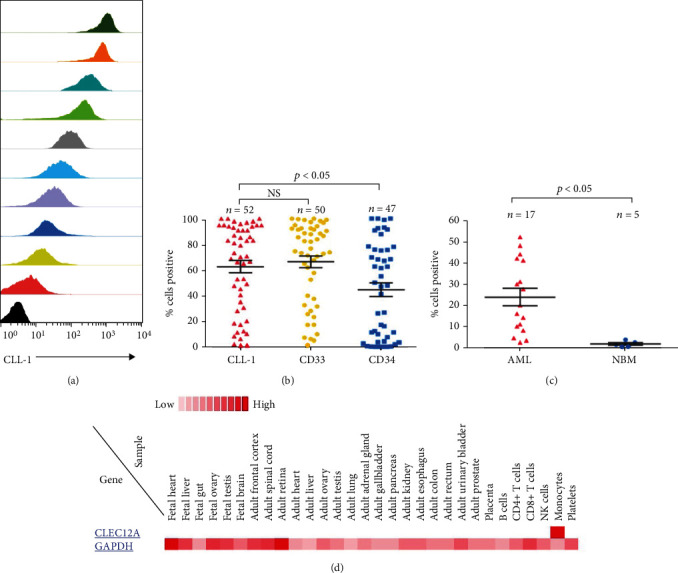
Expression of CLL-1 on AML and stem cells. (a) Variant CLL-1 expression levels among primary AML samples. CLL-1 staining from ten representative samples is shown. (b) Distribution of CLL-1+, CD33+, and CD34+ cells in primary AML samples. (c) CLL-1 expression on CD34+CD38- stem cells in AML and NBM. (d) Expression profile of CLL-1 in normal tissue at protein level was assessed by utilizing publicly available databases for mass-spectrometry proteomic analysis (Human Proteome Map: CLL-1). AML: acute myeloid leukemia; NBM: normal bone marrow; NS: not significant.

To analyze the expression profile of CLL-1 on AML blasts, we defined positive antigen expression as the expression of the antigen in more than 20% of the sample cells. In the prospectively collected test set, 41 of 52 (78.8%) patients stained CLL-1 positive with different intensities, consistent with our previous report [14]. Representative examples of CLL-1 expression on primary AML samples have been shown in Figure 1(a). In most cases, the bulk of the population of blast cells showed no clear separation in populations with positive and negative blasts.

Since CD33 and CD34 were classic markers for AML, we measured the expression of these two markers in combination with CLL-1 on primary AML samples. We found that CD33 and CD34 antigens were expressed on >20% of blast cells in 43 of 50 (86.0%) and 28 of 47 (59.6%) AML samples, respectively. Interestingly, of the 7 CD33-negative samples, 3 were positive for CLL-1 antigen. Furthermore, of the 19 CD34-negative samples, 15 were positive for CLL-1 antigen. When we compared the antigen expression levels, we found that CLL-1 was more frequently expressed than CD34 (*p* < 0.05), but no difference with CD33 (Figure 1(b)).

We next investigated the expression of CLL-1 on CD34^+^CD38^−^ stem cells from part AML patients (*n* = 17) and healthy donors (*n* = 5). We observed that CLL-1 expression on LSCs showed interindividual variability from 2.5% to 52%. And most importantly, the expression level on LSCs was higher than on HSCs (mean, 23.8% versus 1.8%, *p* < 0.05, Figure 1(c)). This suggests that targeting CLL-1 has the potential to eradicate most leukemia blasts including LSCs while sparing the normal HSCs.

Review of protein expression from publicly available databases demonstrated that CLL-1 expression was restricted only to cells of the mononuclear lineage and was not identified on normal tissue from major organ systems (Figure 1(d)).

### 3.2. CLL-1 Expression and Clinicopathological Features

We established the cutoff value of CLL-1 expression at 59.0% with an area under curve (AUC) of 0.694 (*p* = 0.017) (Supplementary Figure 2), and we classified the patients into high and low CLL-1 groups according to this cutoff point. Of the 52 samples, there were 33 CLL-1^high^ (>59.0%) and 19 CLL-1^low^ cases (<59.0%). As seen in Table 1, the CLL-1^low^ group was significantly related to lower BM blast percentage (*p* < 0.05). More importantly, the CLL-1^low^ group was closely related to CR rate which was significantly lower than the CLL-1^high^ group (35.3% versus 73.3%, *p* < 0.05). However, we did not find any significant correlation between CLL-1 expression level and other clinicopathological features including gender, age, disease status, WBC count at diagnosis, FAB subtype, and risk group (data not shown).

### 3.3. CLL-1 Expression and Survival Outcomes

**Figure 2:**
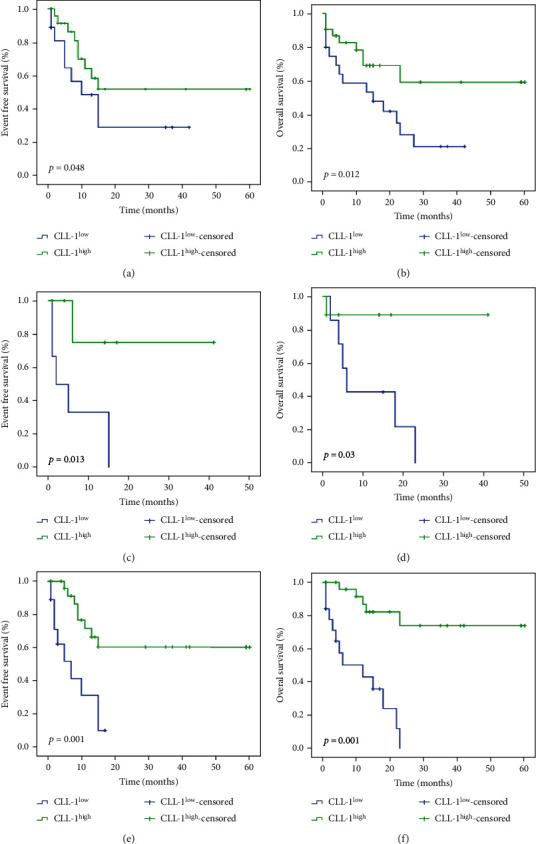
Patient survival. Probability of EFS (a) and OS (b) in AML patients according to CLL-1 expression (entire cohort). EFS (c) and OS (d) in AML patients with poor outcome according to CLL-1 expression. EFS (e) and OS (f) in AML patients according to treatment response. EFS and OS were estimated using the Kaplan-Meier method, and the log-rank test was used for comparison of the survival curves. EFS: event-free survival; OS: overall survival; AML: acute myeloid leukemia; CR: complete response.

After a median follow-up time of 17 months (range, 1-60 months), the median EFS and OS were 15 months (95% confidence interval (CI) 11.9-18.0) and 23 months (95% CI 16.2-29.8), respectively. The estimated 2-year EFS was 43.4% (95% CI 25.6%-61.2%) while the estimated 2-year OS was 42.9% (95% CI 25.5%-60.3%). During the follow-up period, 23 patients died due to disease progression. After having established the definition of CLL-1^high^ and CLL-1^low^, the prognostic relevance of CLL-1 was investigated. As depicted in Figures 2(a) and 2(b)), the lower CLL-1 level group showed significantly inferior EFS (*p* = 0.048) and OS (*p* = 0.012) compared to the higher group. In view of the prevalence of risk-adapted therapy is common in AML nowadays, it was also important to check the possible additional value of CLL-1 in different risk status of AML patients. We found that the CLL-1^high^ patients (*n* = 9) had better EFS than the CLL-1^low^ patients (*n* = 7) in the poor-risk group (*p* = 0.01, Figures 2(c) and 2(d)). Hence, out of these 16 poor-risk patients, CLL-1 could identify and differentiate 9 cases with better prognosis, accounting for 56.3% (9/16) of the patients. In addition, our study also showed that both the EFS (*p* = 0.001) and OS (*p* < 0.001) were significantly better among patients who attained a CR after one cycle of chemotherapy than those without a CR (Figures 2(e) and 2(f)).

### 3.4. CLL-1 Expression with Prognosis

Univariate and multivariate analyses of potential prognostic factors for EFS an OS are summarized in Table 2. Univariate analysis revealed that the significant factor affecting EFS was treatment response (*p* = 0.002), and the significant factors affecting OS included treatment response (*p* < 0.001) and CLL-1 expression (*p* = 0.019). Multivariate analysis showed that the treatment response and CLL-1 expression remained significantly independent factors for OS (*p* = 0.001 and 0.045, respectively). However, the differences in gender (*p* = 0.702 for EFS, *p* = 0.200 for OS), age (*p* = 0.848 for EFS, *p* = 0.159 for OS), WBC counts (*p* = 0.218 for EFS, *p* = 0.820 for OS), BM blasts counts (*p* = 0.146 for EFS, *p* = 0.922 for OS), and risk stratification (*p* = 0.388 for EFS, *p* = 0.395 for OS) were not statistically significant.

## 4. Discussion

AML is a highly heterogeneous disease with different pathophysiological characteristics, therapeutic response, and prognosis [26]. The leukemia-associated immunophenotype varies greatly from patient to patient and is not necessarily stable throughout the course of the disease. In addition, patients with the same cytogenetic risk still have heterogeneity of clinical outcomes [27]. Therefore, it is of great significance to identify supplementary biomarkers for further improving the diagnosis of AML and refining the prognosis of patients with AML. In this study, we detected the expression of CLL-1 in samples of nonselected AML patients and evaluated the clinical outcomes of AML patients according to CLL-1 expression. Our data suggest that CLL-1 is a specific biomarker for AML diagnosis, and the expression of CLL-1 is able to complement the classic markers CD33 or CD34. Furthermore, the abnormal expression of this antigen may be a predictor of inferior outcomes in AML patients, particularly those in poor cytogenetic risk group.

Upon analysis of 52 de novo or relapsed AML samples, we confirmed that CLL-1 was expressed on the cell surface of the majority of AML blasts (78.8%), clearly indicating that the CLL-1 could be used to be the routine FCM in AML. Consistent with previously published work, we found that CLL-1 was also present on the CD34^+^/CD38^−^ LSCs with heterogeneous expression pattern [15], while absent on the normal HSCs. This suggested that CLL-1 aids in discrimination between normal and leukemic stem cells. Besides, given the frequent expression on AML blasts and LSCs, yet limited expression on nonhematopoietic tissues and HSCs presented here and in previous reports [13–15, 28], CLL-1 may be one of the promising surface target molecules for AML. Up to now, multiple strategies have been adopted that based on unconjugated monoclonal antibodies [12], antibody-drug conjugates [16, 17], bispecific antibodies [18–20], and chimeric antigen receptor- (CAR-) engineered T cells (CAR-T cells) [14, 22, 23] in preclinical studies. There was only one CLL-1×CD3 bispecific antibody named MCLA-117 had entered the clinical trial, which had recruited primary or secondary AML in old patients (≥ 65 years) with high-risk cytogenetics or intolerance of induction therapy since 2016 (ClinicalTrials.gov ID: NCT03038230) [21]. We have previously developed a third-generation CAR-T cell targeting CLL-1 which showed potent antitumor activity both in vivo and in vitro [14].

How would CLL-1 perform compared with the classic markers CD33 and CD34? In the present study, we found that CLL-1, CD33, and CD34 were positive in 78.8%, 86.0%, and 59.6% of AML samples, respectively. Interestingly, CLL-1 was complemented with CD33 or CD34 as diagnostic markers and (potential) therapeutic targets, because 42.9% of the CD33 negative AML samples expressed CLL-1, and 78.9% of the CD34 negative samples expressed CLL-1. Moreover, when the CLL-1 and CD33 were combined, 92% (46 of 50) of the AML samples could be identified. Consequently, our coexpression analysis suggested that combinatorial detecting approaches might enhance diagnosing efficiency in AML, and combinatorial targeting approaches might enhance therapeutic efficacy in AML that should be validated in the future. Besides, CD34, a stem cell-specific marker in AML, is widely used to monitor minimal residual disease (MRD). The addition of CLL-1 maybe valuable in increasing the likelihood of upfront MRD marker identification by FCM in the CD34^−^ subgroup. Notably, we are the first to compare the antigen expression levels of CLL-1, CD33, and CD34 simultaneously on AML bulk cells.

We next analyzed the relationship between CLL-1 expression level and clinicopathological features. We found that CLL-1 was nonrandomly expressed on AML samples throughout the different FAB subtypes and risk groups. After having established the definition of CLL-1^high^ and CLL-1^low^ according to cutoff 59.0%, we found that the low expression level of CLL-1 was significantly correlated with lower BM blast percentage, and what is more, the CR rate after cycle 1 of the CLL-1^low^ group was significantly lower than the CLL-1^high^ group (*p* < 0.05). We know that low remission rates often indicate poor prognosis, and we did demonstrate that both the EFS and OS were significantly worse among patients who did not reach CR than those did. Meanwhile, no CR was demonstrated to be an independent factor associated with EFS and OS in multivariate Cox regression model. However, a statement on the possible correlation with other clinicopathological features, such as gender, age, disease status, WBC count at diagnosis, FAB subtype, and risk group, cannot be presently made probably due to the relatively small number of patients.

When we compared the survival between CLL-1^high^ and CLL-1^low^ groups, we discovered that EFS and OS of the CLL-1^low^ group were significantly lower than the CLL-1^high^ group (*p* < 0.05). Furthermore, in univariate Cox regression model analysis, we found that the CLL-1^low^ group was an independent prognostic value associated with OS, and multivariate Cox regression model analysis showed that CLL-1^low^ was still independent from other well-established factors. However, the low expression level of CLL-1 did not maintain its value in predicting EFS. Taking into consideration that risk stratification is critical in AML, we analyzed the impact of CLL-1 expression in each risk subgroup. The results showed that the CLL-1^high^ patients had better EFS and OS than the CLL-1^low^ patients (*p* < 0.05) in the poor-risk group. This implied that CLL-1^high^ patients in the poor-risk group had a more intermediate prognosis comparing to poor-risk CLL-1^low^ patients, who actually have a worse prognosis. As a result, the CLL-1 expression should be incorporated into future risk-adapted therapy and prognosticating relapse risk in this subset of poor-risk AML patients. We did not find similar results in other risk subgroups probably due to the small numbers. This study agreed with Wang et al., who also demonstrated that CLL-1^low^ indicated poor prognosis (*p* < 0.001 for EFS and OS) in patients with AML. Nevertheless, they detected the CLL-1 expression only in de novo CD34^+^ Non-M3 AML, while we evaluated the CLL-1 expression in nonselected patients including de novo and relapsed AML, further supporting its high value in prognosis. Besides, some studies have compared the expression of CLL-1 on AML samples at diagnosis, treatment, and relapse and found no significant difference [13, 15, 29–31]. This suggested that CLL-1 could be used as a reliable marker for disease follow up/detection of MRD.

Concerning the mechanism of low CLL-1 expression in leukemic blasts as a predictor of poor prognosis, it remains unclear. It was previously reported that CLL-1 might play a role in the control of cell maturation [7], so we consider that the loss of CLL-1 expression may prevent leukemia cell proliferation and keep it in a relatively static state, thus reducing the sensitivity to chemotherapy. In addition, we discovered that the proportion of CLL-1low group in poor-risk stratification was higher than that of CLL-1high group, which may also partly explain the poorer prognosis of patients with low CLL-1 expression. However, further studies will be required to understand the mechanism underlying the impact of CLL-1 expression for prognosis.

Taken together, we have demonstrated that CLL-1 is one of the promising surface molecules for AML diagnosis; meanwhile, CLL-1 is easy to measure in clinical practice and thus can be incorporated into the routine practice of most clinical laboratories. Furthermore, we have proved that CLL-1 is an effective tool to predict the survival of AML patients, so it can be used as a supplement to the current AML prognostic risk stratification system and may optimize the clinical management of AML. However, the relatively small number of patients and the short follow-up time limited us to draw a robust conclusion in our study, and cytogenetic and molecular genetic profiles were not complete for all patients. Further and larger-scale studies are required to more clearly define the significance of CLL-1 expression in AML and also to elucidate the underlying mechanisms in the future.

## 5. Conclusions

In summary, we report that CLL-1 was expressed on the cell surface of the majority of AML blasts (78.8%) and also expressed on leukemic stem cells in varying degree but absent on normal hematopoietic stem cells. Notably, CLL-1 was able to complement the classic markers CD33 or CD34. In addition, we discovered that EFS and OS of the CLL-1^low^ group were significantly lower than the CLL-1^high^ group, and low CLL-1 expression seems to be independently associated with shorter OS. These results suggested that CLL-1 may serve as a biomarker for diagnosis and prognosis of AML.

## Figures and Tables

**Table 1:** Patient characteristics.

Characteristic	CLL-1^low^ patients	CLL-1^high^ patients	*p* value
Total, no. (%)	19 (36.5)	33 (63.5)	
Gender, no. (%)			0.76
Male	11 (57.9)	17 (51.5)	
Female	8 (42.1)	16 (48.5)	
Age at diagnosis, years, median (range)	38.0 (13-75)	50.0 (7-75)	0.44
Disease status, no. (%)			0.26
De novo	14 (73.7%)	29 (87.9%)	
Secondary	5 (26.3%)	4 (12.1%)	
WBC count at diagnosis, 10^9^/L, median (range)	17.3 (0.5-345.6)	31.7 (0.8-373.9)	0.33
Bone marrow blasts at diagnosis, %, median (range)	30.0 (10.1-91.5)	73.0 (9.5-96.0)	0.03
FAB classification, no. (%)			0.39
M1	2 (10.5)	1 (3.0)	
M2	5 (26.3)	9 (27.3)	
M3	1 (5.3)	4 (12.1)	
M4	0 (0)	3 (9.1)	
M5	9 (47.4)	8 (24.2)	
M6	0 (0)	1 (3.0)	
M7	0 (0)	0 (0)	
Not done	2 (10.5)	7 (21.2)	
Risk group, no. (%)			0.44
Favorable	1 (5.3)	2 (6.1)	
Intermediate	3 (15.8)	11 (33.3)	
Unfavorable	7 (36.8)	9 (27.3)	
Not done	8 (42.1)	11 (33.3)	
CR reached after cycle 1, no. (%)			0.03
Yes	6 (35.3)	22 (73.3)	
No	11 (64.7)	8 (26.7)	
Allogeneic stem cell transplantation, no. (%)			1.00
Yes	2 (10.5)	5 (15.2)	
No	17 (89.5)	28 (84.8)	

CLL-1: C-type lectin-like molecule-1; WBC: white blood cells; FAB: French-American-British; CR: complete response.

**Table 2:** Univariate and multivariate survival analysis.

Clinical factors	Event-free survival		Overall survival			
	Univariate		Univariate		Multivariate	
	*p*	HR (%95 CI)	*p*	HR (%95 CI)	*p*	HR (%95 CI)
Gender (male)	0.702	0.834 (0.329-2.115)	0.200	1.757 (0.743-4.159)	—	—
Age (>50)	0.848	0.911 (0.351-2.362)	0.159	1.817 (0.792-4.167)	—	—
WBC (>100∗10^9^/l)	0.218	0.036 (0.000-7.056)	0.820	0.890 (0.325-2.435)	—	—
BM blasts (>50%)	0.146	0.494 (0.191-1.278)	0.922	0.960 (0.422-2.186)	—	—
Risk stratification (poor)	0.388	1.383 (0.662-2.889)	0.395	1.407 (0.640-3.094)	—	—
CLL-1 (<59%)	0.061	2.423 (0.960-6.111)	0.019	2.738 (1.183-6.339)	0.045	2.815 (1.021-7.758)
No CR after cycle 1	0.002	4.332 (1.687-11.119)	<0.001	7.483 (2.571-21.780)	0.001	6.773 (2.228-20.587)

HR: hazard ratio; CI: confidence interval; WBC: white blood cells; BM: bone marrow; CR: complete response.

## Data Availability

The data used to support the findings of this study are available from the corresponding author upon request.

## Supplementary Materials

Supplementary Materials Supplementary Figure 1: the gating strategy of AML blasts and LSCs. (A) Initially, cells were gated based on forward and side scatter properties. Subsequently, AML blasts were selected based on low-side scatter versus CD45^dim^ expression. (B) After AML blasts were selected, CD34^+^ cells were then gated. Finally, CD38^−^ cells were recognized as LSCs. AML: acute myeloid leukemia; LSC: leukemic stem cells. Supplementary Figure 2: the ROC curve analysis for the optimal cut-off point of CLL-1 expression. The most discriminative cut-off value for CLL-1 was 59% with a sensitivity of 60.9% and a specificity of 79.4%; the area under ROC curve is 0.694 (*p* = 0.017).
