# Supplementary material for: C-Type Lectin-Like Molecule-1 as a Biomarker for Diagnosis and Prognosis in Acute Myeloid Leukemia: A Preliminary Study
Source: Biomed Res Int. 2021 Mar 11;2021:6643948. doi: 10.1155/2021/6643948 (PMC7979301; doi:10.1155/2021/6643948)
Supplement: Supplementary Materials — Supplementary Figure 1: the gating strategy of AML blasts and LSCs. (A) Initially, cells were gated based on forward and side scatter properties. Subsequently, AML blasts were selected based on low-side scatter versus CD45dim expression. (B) After AML blasts were selected, CD34+ cells were then gated. Finally, CD38− cells were recognized as LSCs. AML: acute myeloid leukemia; LSC: leukemic stem cells. Supplementary Figure 2: the ROC curve analysis for the optimal cut-off point of CLL-1 expression. The most discriminative cut-off value for CLL-1 was 59% with a sensitivity of 60.9% and a specificity of 79.4%; the area under ROC curve is 0.694 (p = 0.017). [file 6643948.f1.docx]

**Supplementary** **FIGURE 1**

**
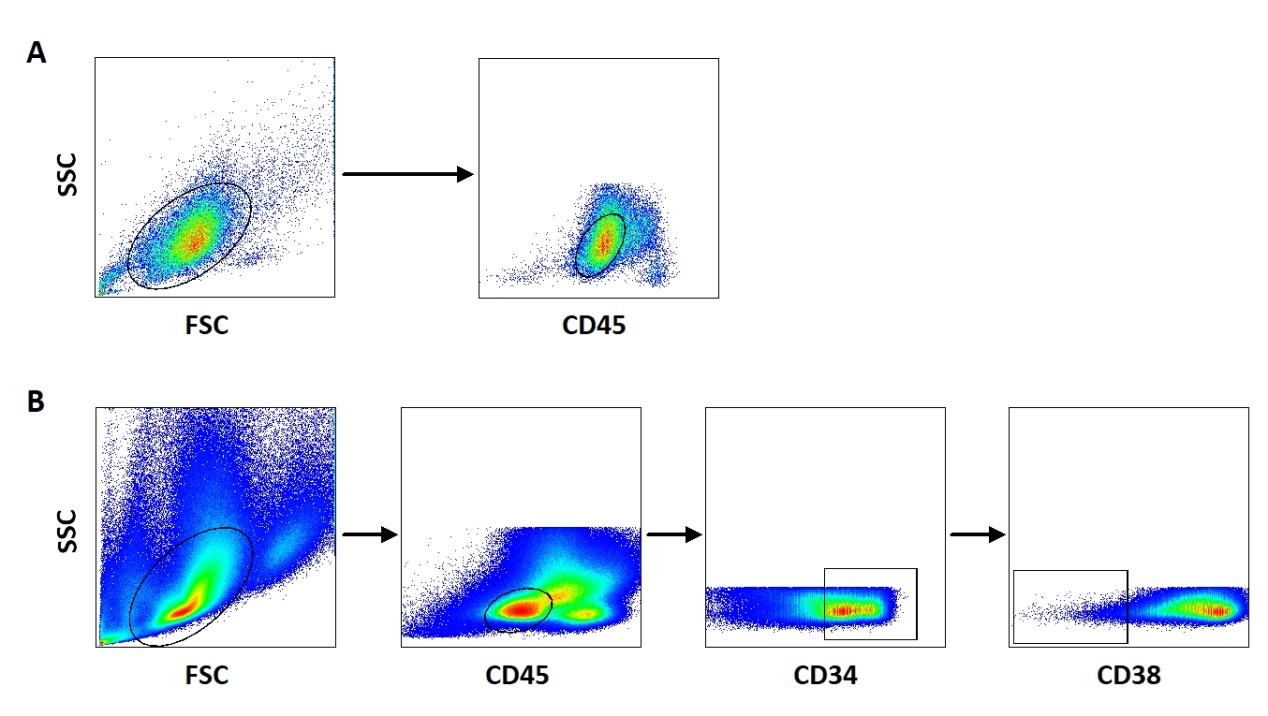
**

Supplementary FIGURE 1: The gating strategy of AML blasts and LSCs. (A) Initially, cells were gated based on forward and side scatter properties. Subsequently, AML blasts were selected based on low side scatter versus CD45^dim^ expression. (B) After AML blasts were selected, CD34^+^ cells were then gated. Finally, CD38^-^ cells were recognized as LSCs. AML: acute myeloid leukemia; LSC: leukemic stem cells.

**Supplementary** **FIGURE 2**


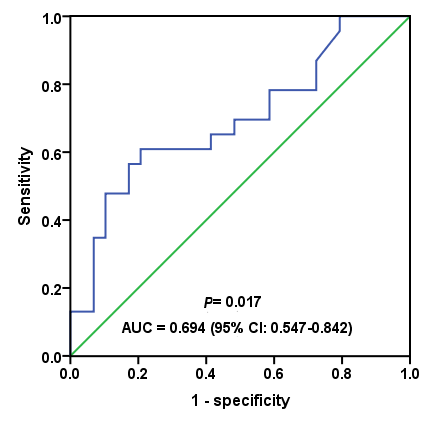


Supplementary FIGURE 2: The ROC curve analysis for the optimal cut-off point of CLL-1 expression. The most discriminative cut-off value for CLL-1 was 59% with a sensitivity of 60.9% and a specificity of 79.4%, the area under ROC curve is 0.694 (*p* = 0.017).
